# Nanoprofilometry study of focal conic domain structures in a liquid crystalline free surface

**DOI:** 10.3762/bjnano.8.254

**Published:** 2017-11-29

**Authors:** Anna N Bagdinova, Evgeny I Demikhov, Nataliya G Borisenko, Sergei M Tolokonnikov

**Affiliations:** 1 Cryogenic Department, P.N. Lebedev Physical Institute of the Russian Academy of Sciences, 53 Leninskiy Prospekt, Moscow, 119991, Russia,; 2 Neutron Physics department, P.N. Lebedev Physical Institute of the Russian Academy of Sciences, 53 Leninskiy Prospekt, Moscow, 119991, Russia

**Keywords:** focal conic domains, free boundary, liquid crystals, nanoprofilometer, smectic-A phase

## Abstract

This work presents the first high-resolution nanoprofilometry study consisting of nanoscale resolution surface profile measurements and high-quality visualization of a the free surface of a liquid crystal–air boundary. The capabilities of this new experimental method, as applied for liquid crystal free boundaries, are discussed. The formation of focal conic domain structures at the smectic-A–air free boundary was detected and studied.

## Introduction

The free surface of liquid crystals has been a subject of great interest since the beginning of liquid crystal science. Liquid crystalline free boundary research is very important because it shows that the intrinsic free surface properties are not influenced by the substrate anchoring [1–2]. This understanding is important for display-quality technology and production enhancement. The study of the free liquid crystalline boundary is interesting for applications such as liquid crystalline colors and coatings as well as cosmetics. To study liquid crystalline free boundary structures, common nanotechnology tools are used, for example atomic force microscopy (AFM) [3], light reflection, high-resolution microscopy, X-ray reflection, and transmission electron microscopy. Nanoprofilometers have shown great progress in the last years and are now capable of resolving dimensions of several nanometers on the surface. Nanoprofilometers, or surface analyzers, have been used for technology control in micro- and nanoelectronics to control the production output. In this paper we report the first application of a nanoprofilometer for analysis of the free surface of liquid crystals.

In this work, we studied structures in samples with one free surface. The second boundary was covered by some orienting material with strong boundary conditions. In this case, focal conic domain structures can occur in smectic-A phase [4–8]. Here, the smectic sample would have a tendency to break uniformity and form cavities. In liquid crystal (LC) displays this can lead to defects on large screens.

Interesting attempts to apply profilometry to liquid crystalline surfaces has been previously undertaken [9–10]. Although one of such profilometers [9] had high precision, this was one order of magnitude less than the results obtained with the interferometric surface structure analysis work of this paper. In another [10], the sample became solid after evaporation of the liquid part of the suspension. In both cases no visual images of the surface were demonstrated.

## Results

The free surface structure of the liquid crystal compound 8CB was studied using a nanoprofilometer (ZYGO, NewView 6K). The optical scheme and principal of operation of the microscope are presented in the Experimental section of this paper.

The LC compound 4-*n*-octyl-4’-cyanobiphenyl (K24 or 8CB) has the following liquid crystalline phase sequence: isotropic (41 °C), nematic (32 °C), smectic-A (22.2 °C).

**Figure 1:**
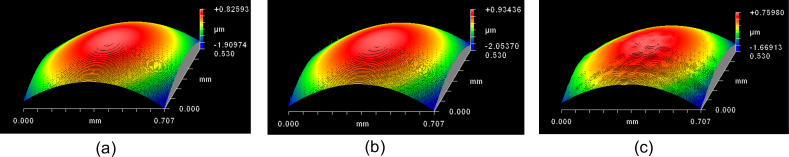
Interferometric surface structure analyzer (ISSA) study of the liquid crystal 8CB free surface in (a) isotropic liquid *T* = 43 °C, (b) nematic *T* = 37 °C and (c) smectic *T* = 32 °C phases on the LC display substrate.

The profilometer is an interferometric surface structure analyzer (ISSA) that allows three dimensional images of the surface structures to be obtained. Its vertical scan range is 150 μm, the vertical resolution is less than 0.1 nm, and the lateral resolution depends on the objective. We used a 20× objective with a lateral resolution 0.87 μm. Figure 1 shows the 8CB free surface ISSA scans for different liquid crystalline phases. Figure 1a shows the structure of the free surface 8CB in isotropic phase measured by ISSA at *T* = 43 °C. The surface is smooth and possesses no peculiarities. The thickness of the LC layer in the top part of the droplet was 109 μm. Figure 1b demonstrates the structure of the free surface in the nematic phase at *T* = 37 °C. It looks similar with an isotropic phase and does not reveal any structures on the surface. The thickness of the LC layer in the top part of the droplet was 114 μm. Figure 1c demonstrates the beginning of the formation of crater-like structures on the free surface in the smectic phase at *T* = 32 °C on the LC display substrate. The depth of the craters is about 0.1 μm.

For the next experiments, the 8CB materials was heated up to 43 °С – the temperature of the isotropic liquid phase, and then cooled down to the smectic-A (SmA) phase temperature.

**Figure 2:**
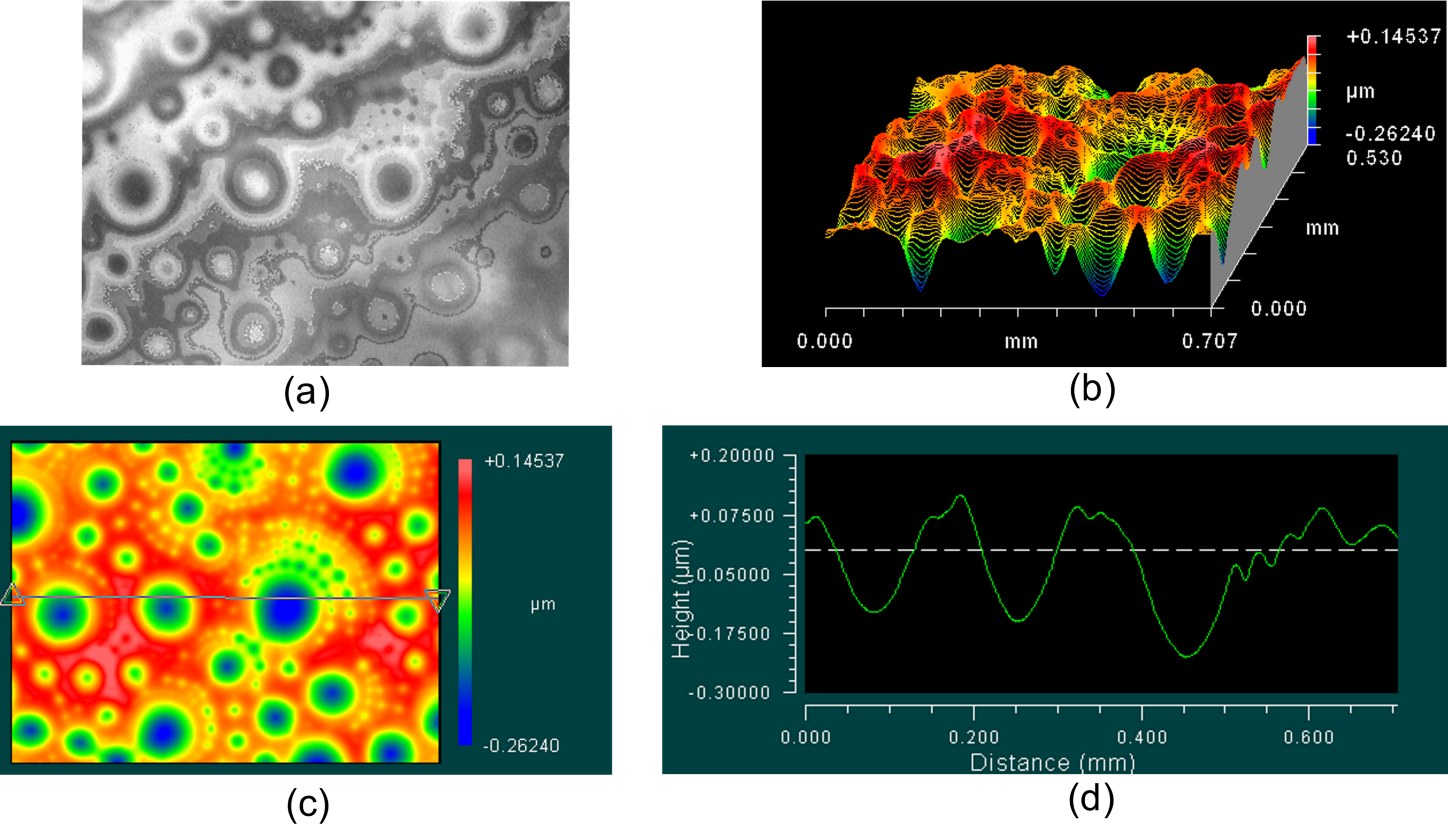
Interferometric surface structure analyzer (ISSA) study of the liquid crystal (LC) 8CB free surface in smectic-A phase at *T* = 30 °C on the LC display substrate. The maximal thickness of the droplet in the top is 53 μm. (a) demonstrates a real view of the surface structure, (b) is a 3D reconstruction, (c)shows the top of view of (b), and (d) is a line profile section along the line in (c).

Figure 2 shows the process of formation and relaxation of the craters over the course of 10 min at 30 °С in the smectic phase. The after 10 min, no further change of the crater shape was observed. The sample was cooled at a rate of 0.5 °C/min starting from isotropic liquid.

Figure 2a shows the real view of the surface structure and demonstrates that there are a significant number of crater-like structures with different sizes and depths. A 3D reconstruction of the surface is represented in the Figure 2b and its top view in Figure 2c. The range of the depth from the lowest to the highest part (peak-to-valley) is 408 nm. Figure 2d shows a line profile along the line in Figure 2c, where the depth of the biggest crater in this cross-section is 340 nm.

Experiments with different film thickness showed that the size of the crater-like structures become smaller as the film thickness decreases. The films thickness can be reduced by spreading the substance with a razor blade. At a thickness of about 5 μm, the profilometer was not able register a reasonable picture because of the lack of back-scattered light intensity.

At thicknesses of less than 5 μm, a change in the shape of the sample takes place: a uniform surface has the tendency to break into smaller islands. This leads to an increase in the light scattering and a decrease in the intensity of the back reflection of light. This is the reason that a stable interference pattern could not be established.

**Figure 3:**
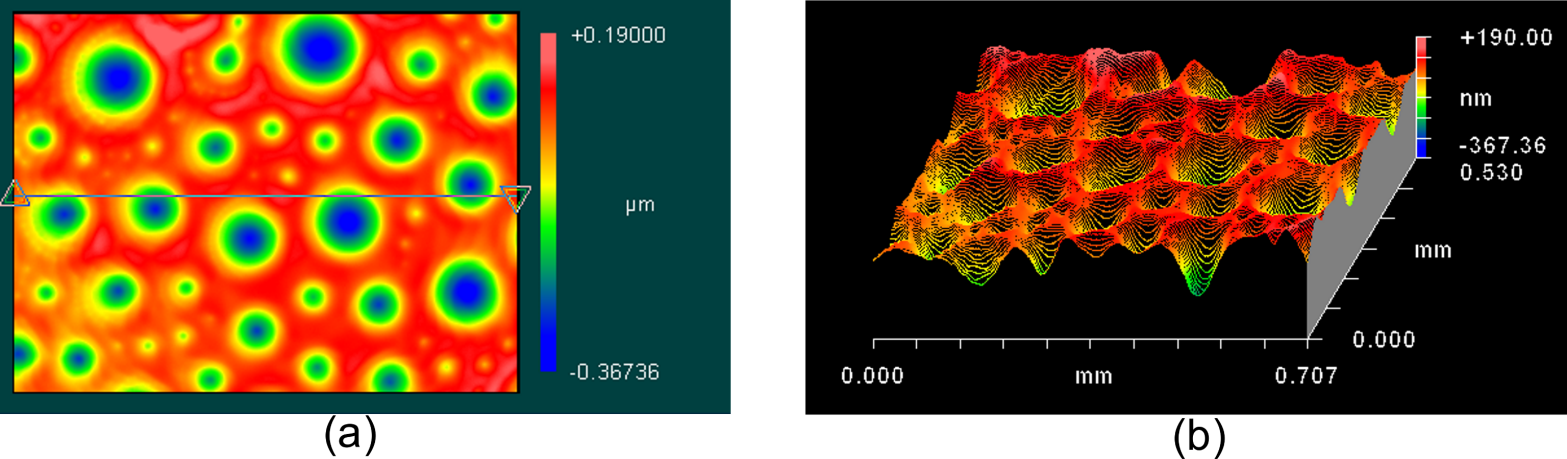
Interferometric surface structure analyzer (ISSA) image of the liquid crystal 8CB free surface in smectic phase at *T* = 28 °С on the glass substrate, a (a) 3D and (b) 2D view. The peak-to-valley height is 557 nm.

Figure 3 demonstrates a 8CB smectic-A phase surface at 28 °С on the glass substrate. Before use, the quartz glass substrate was cleaned with an alcohol mixture and dried. In order to get more detailed information about surface inhomogeneity, the sphericity of a droplet can be extracted by the program software MetroPro by ZYGO as shown on Figure 3.

A reverse experiment was performed by heating the films and showed that the crater structures of 8CB in smectic phase become smaller than at 32 °C and disappear after the phase transition of smectic-A–nematic.

**Figure 4:**
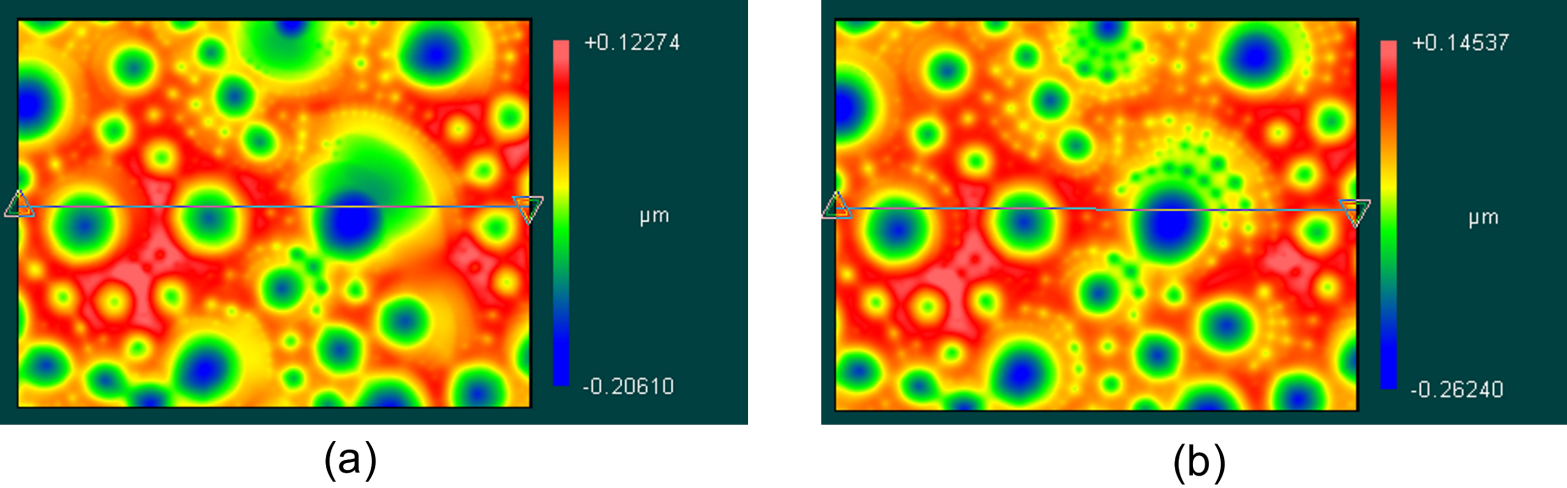
Interferometric surface structure analyzer (ISSA) study of the liquid crystal 8CB free surface in smectic phase at *T* = 30.5 °C. (a) The peak-to-valley depth was 329 nm and (b) after 15 min, the peak-to-valley depth was 408 nm.

The dynamics of the crater formation is shown on Figure 4. In this experiment, a small droplet of the 8CB material was placed on the LC display substrate with a sharp tip. Then the substance was heated to the temperature of the isotropic liquid phase 43 °С on a heating stage. Then the heating table was turned off and the substance was cooled to room temperature. At a temperature of 32 °С, the phase transition to the smectic phase occurred and the crater formation began. The temperature was stabilized at 30.5 °C. Figure 4 illustrates the structure in smectic phase at 30.5 °C. Figure 4a,b demonstrates the appearance of small crater-like structures around larger ones and that the average depth changes from 329 nm (Figure 4a) to 408 nm (Figure 4b) after 15 min of relaxation.

## Discussion

8CB is a classic material for observation of focal conic domains (FCDs). FCDs appear when two competing boundary conditions take place at a boundary. In our case, the liquid crystal has strong boundary conditions on the solid substrate and the director is oriented parallel to the substrate. On the free boundary, the stable director orientation is perpendicular to the air–liquid crystal boundary. Such a case has been described in several papers [11–17] and the stable director field structure has been calculated in the SmA phase. The pattern of the SmA free boundary shown in of Figures 2–4 is similar to the experimental observations in [3–8] and calculations in [8,11–14]. Therefore, the crater patterns observed on Figures 2–4 are considered to be FCD structures on the free surface.

The nature of the SmA crater is associated with competition of the high-energy substrate adhesion and preferred orientation of the SmA director parallel to the solid boundary and stable perpendicular director orientation to the free surface. This difference leads to the observation of “conic holes” on the boundary. The FCD structure is a stable director field configuration and defines the elastic moduli of liquid crystals. The shear modulus, *G*, is given as

[1]
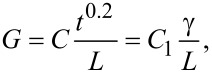


where *C* and *C*_1_ are scaling constants depending on surfactant, *t* is the relative temperature, γ is the surface tension coefficient of the free surface with FCDs, and *L* is the FCD dimension.

This relation underlines that the smectic-A phase with FCDs has different elastic properties compared to an ideal smectic-A sample. Usually, it is observed that for the shear parallel to the smectic layers, the shear modulus is zero. In the case of a SmA sample with a FCD, the free sliding is hindered by the presence of FCDs. This problem is discussed in detail in [4]. Equation 1 is valid in our case because we have a system of FCDs in smectic-A phase. This statement qualitatively corresponds to our results because we observe a slight increase of the FCD depth upon decreasing temperature. The elasticity of the smectic-A liquid crystal is physically related to the domain size and surface tension. This is very important for the analysis of the stable director field configurations and mechanisms behind their destruction in LC displays. Even in the case of a homogeneous director field orientation on solid substrates, FCDs can occur inside of displays when the director field orientation is perpendicular to the display substrate. This configuration is normally unstable and relaxes back after the electric field is switched off. But in some cases, due to the electric properties, the director field can overcome the corresponding energy barrier and the display can lose orientation over a large area, initiating the formation of FCDs inside of the liquid crystalline layer. In the case of inner boundary formation due to dislocations, the nanoprofilometer technique can be applied for structure observation. The FCD structures can be compared with twist grain boundary (TGB)-like structures [18–20] because of some interdomain regions, which can explain the holes in the FCD-structured surface.

We could expect some pattern formation starting from the nematic phase. The surface of the isotropic liquid and the nematic phase of 8CB revealed no structures and was found to be isotropic in both cases. The FCDs are formed just below the phase transition SmA–nematic. It is important to underline that the FCDs are a result of the correlation of the director field orientations on both surfaces. The interaction between surfaces due to the conic domain structures is important for thicknesses of about 1–2 μm.

## Conclusion

In this paper we report the first nanoprofilometry study of the free boundary in isotropic liquid, nematic and smectic-A phases of the liquid crystalline material 8CB. We observed FCD structures in the smectic phases. Comparison of our study with other contributions on FCDs shows good quantitative correlation of the dimensions of the domains. This is an important result for nanoprofilometer calibration because it delivers calculated images. The FCD properties qualitatively correlate with theoretical predictions of [8]. Comparing the ISSA method with the other surface-sensitive methods, we can see that ISSA is more powerful as compared to the common microscope interferometer [21–22]. In ISSA we see a reconstructed surface of the sample with very high precision due to the software. ISSA pictures are expected to be consistent with focal conic microscopy images, which will be explored in our publications to follow. It is interesting to compare ISSA images with scanning probe microscopy images (e.g. AFM and SNOM). We expect that the difference between these images will be significant because of the interaction of the tip and the LC surface.

## Experimental

**Figure 5:**
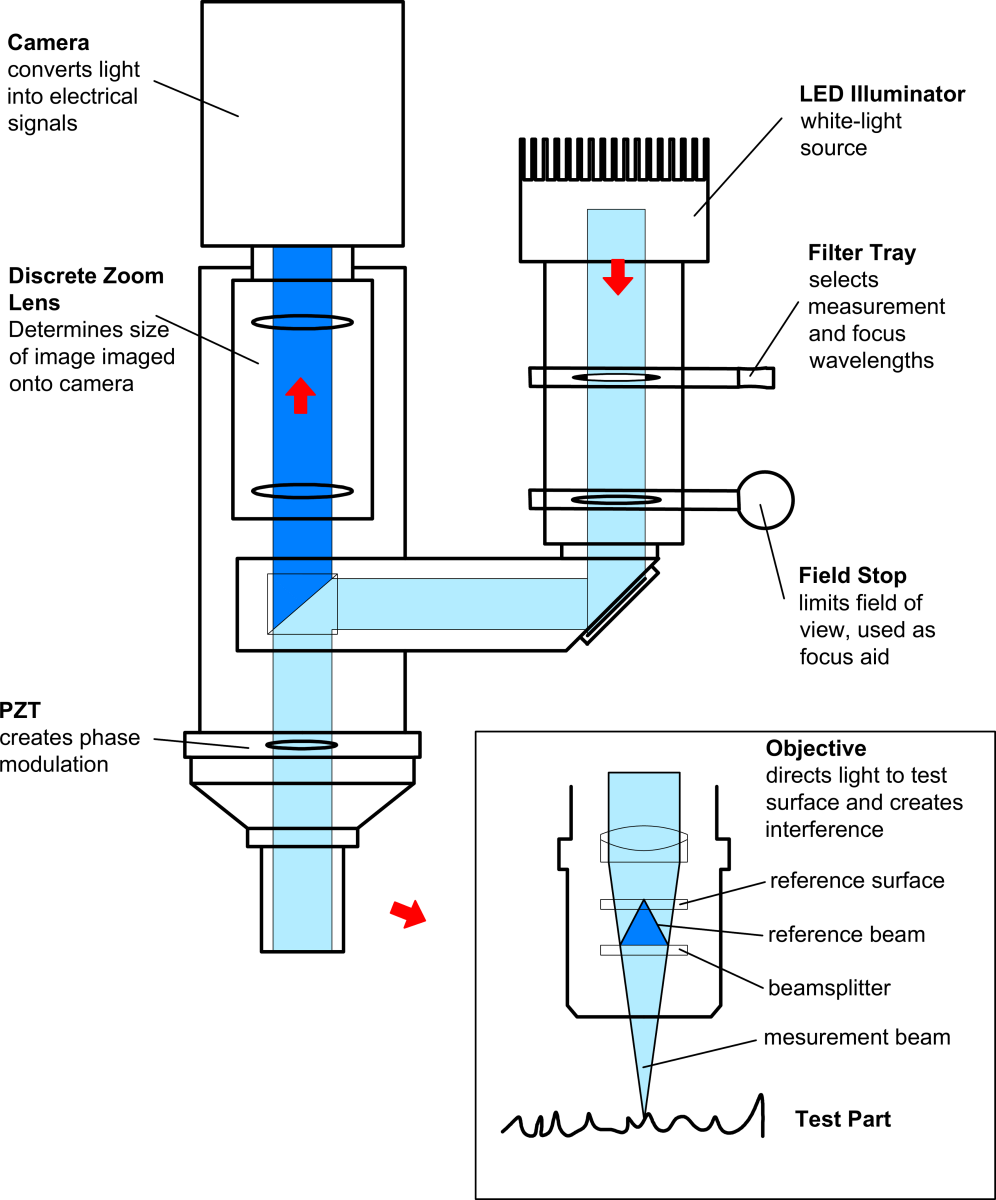
Schematic optical diagram of the interferometric surface structure analyzer (ISSA).

As was previously mentioned, the free surface structure of the liquid crystal compound 8CB has been studied using a profilometer. Scanning interferometry of white light was used in ISSA to obtain images and to calculate and analyze surface structures of the test parts. Light from the microscope is divided in the interferometric objective: one part reflects from the test part and another one reflects from the internal reference surface in the objective. Both parts are then directed to the solid-state camera. A schematic diagram of the ISSA system is shown in Figure 5.

The result of the interference of the two wavefronts is an image of light and dark fringes that indicate surface structure. The test part is scanned by vertical movement of the objective with a piezoelectric transducer (PZT). The intensity in each camera pixel is fixed by video camera and converted to an amplitude by the software MetroPro.

During the experiments, a small droplet of the substance was placed on a piece of LC display substrate with a sharp metallic tool and then put on the heating table. The temperature of the isotropic liquid phase was set and structure of the droplet was observed using ISSA. During heating, the droplet sometimes moved on the surface and it was necessary to wait for the motion to stabilize. In some experiments, the droplet was spread very thin on the substrate with a razor blade pre-heated up to the temperature of the isotropic liquid.

**Figure 6:**
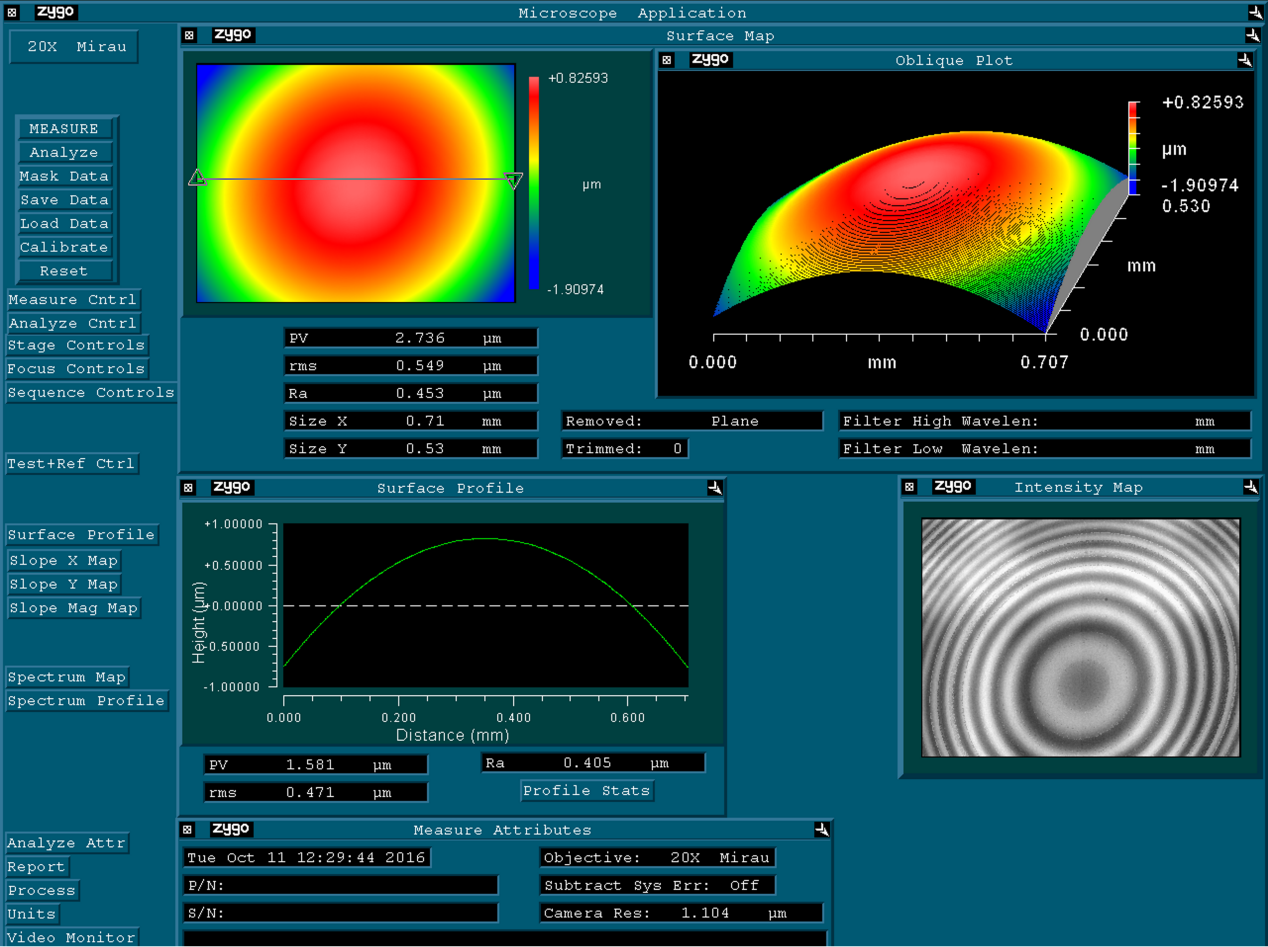
Computer software interface with experimental results of an ISSA scan of the 8CB free surface in the isotropic phase at *T* = 43 °C on the substrate, displayed with strong boundary conditions.

For measuring the thickness of a layer of the liquid crystal substance, the position of the measurement plane was determined by focusing on the outer surface of the object and focusing on the substrate (the transparency of the object made it possible to focus on the substrate surface). The difference between these positions gives us the thickness of the object. The results of the experiment are displayed the computer interface, as demonstrated in Figure 6.

In the right-bottom panel of Figure 6, an interference pattern of the LC film before the calculations is shown in real time. The software enables the 3D reconstruction of the surface. Vertical measurements (normal to the surface) are carried out interferometrically. Horizontal measurements (in the plane of surface) are made by calculating the size of the pixel in the field of the objective. Using these methods, ISSA analyzes and calculates the topography of the surface. The final result is displayed as the three-dimensional color image as shown in the right-top part of Figure 6.

In the left-top part of Figure 6, a two-dimensional top projection is shown. The table near it represents image parameters such as peak-to-valley depth, root-mean-square value, ratio average, size along the *x*-direction, and size along the *y*-direction. The left-bottom part shows a spatial coordinate dependence of the surface profile and represents numerical peak-to-valley data, ratio average, and root-mean-square for this plot.

**Figure 7:**
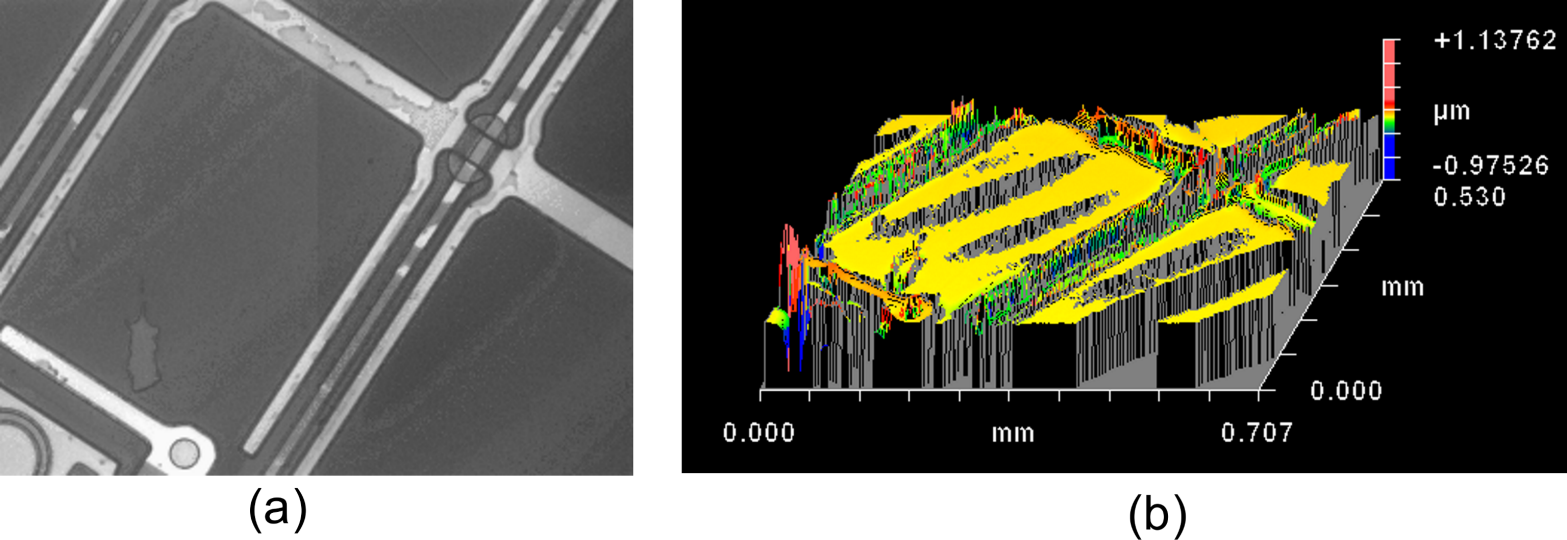
Liquid crystal display substrate (a) without any material and (b) with a very thin liquid crystal film.

As the substrate, a rectangular piece of a Samsung PC liquid crystal display was used (Figure 7). For this, we have broken a commercial Samsung PC display, cleaned and dried it. The LC PC display substrate brings two main advantages. First, it has a large anchoring energy for the liquid crystal director field and it makes for better conditions for FCD formation. Second, it helps to measure the films thickness: we can accurately focus the microscope on the electrodes pattern of the substrate at the bottom edge of the liquid crystal layer, and after that, we focus on the top part of the LC film and determine the thickness.

For comparison, we used a glass substrate without electrodes. On the glass substrate the same surface structures were observed. It is important to note that the FCDs are not observed on the smectic-free surface. The conditions for FCD formation are given in [4]. The nanoprofilometer presented in this work is a noncontact method which can be applied to study not only solid but also LC surfaces as has been demonstrated in this work.
